# MTX Osteopathy Versus Osteoporosis Including Response to Treatment Data—A Retrospective Single Center Study Including 172 Patients

**DOI:** 10.1007/s00223-024-01290-5

**Published:** 2024-09-25

**Authors:** Felix N. von Brackel, Jonathan Grambeck, Florian Barvencik, Michael Amling, Ralf Oheim

**Affiliations:** Department of Osteology and Biomechanics, University Medical Center Hamburg-Eppendorf, Lottestrasse 59, 22529 Hamburg, Germany

**Keywords:** MTX, MTX osteopathy, Stress fracture, Therapy, Osteoporosis, HR-pQCT

## Abstract

MTX is an effective and widely used immunomodulatory drug for rheumatoid diseases. MTX osteopathy is a very rare and specific side effect, characterized by stress fractures at multiple locations in the lower extremity, hampering the patient’s mobility by pain and loss of function. In clinical practice, osteoporosis and MTX osteopathy are repeatedly confused and a comparative workup is needed to clarity it’s specifics. Furthermore, specific treatment options for MTX osteopathy need to be established. We compared patients suffering from MTX osteopathy to patients with osteoporosis (OPO). Patients underwent an extensive clinical workup including blood sampling, bone mineral density measurements, high-resolution peripheral quantitative computed tomography and muscular performance testing. Furthermore, treatment regimes in MTX osteopathy were compared with respect to regain of mobility and pain reduction. 83 patients with MTX osteopathy and 89 with OPO were included. Patients with MTX osteopathy did exhibit fractures predominantly at the lower extremity and pain scores were significantly higher (MTX: 6.75 ± 1.86 vs. OPO: 3.62 ± 2.95, p < 0.0001). MTX-caused mobility restriction was successfully reduced by treatment only if MTX was discontinued (pre-treatment: 2.16 ± 1.19 vs. post-treatment: 1.04 ± 0.87, p < 0.0001). Most mobility gain was achieved by involving anabolic treatment (anabolic: 2.1 ± 1.02 vs. antiresorptive: 1.09 ± 0.94, p < 0.05). In summary, MTX osteopathy is characterized by distinct lower extremity stress fractures leading to severe pain and immobility. Discontinuation of MTX is essential to enable treatment success and involving anabolic treatment seems to be more effectively in mobility regain as antiresorptive treatment alone.

## Introduction

Methotrexate (MTX) emerges as a pivotal pharmaceutical agent with a rich history dating back to the early 1970s. Originally developed for oncological applications, MTX use has undergone a paradigm shift. In today’s medical practice, MTX assumes a central role as a primary disease-modifying antirheumatic drug (DMARD), frequently deployed as a first-line treatment for various rheumatic conditions. Its immunomodulatory properties render it effective in impeding the progression of diseases such as rheumatoid arthritis (RA).

However, MTX also has adverse effects. A bone specific side effect of MTX is known since 1970 [1] and was described as “osteoporotic fractures” and “severe osteoporosis with associated fractures” in growing children [1]. Similar pattern, linked to MTX, have been seen in adults in 1973 [2]. Over time, numerous case reports have increasingly provided evidence that a MTX specific osteopathy is indeed one side effect of MTX therapy, not only in children in oncologic dosages but also in adults using low-dose MTX for rheumatoid diseases [2–11]. MTX osteopathy describes a rare systemic side effect of MTX on bone metabolism leading to low turnover and consequently stress fracture(s), primarily of the lower extremity [3], which occur without adequate trauma causing mechanical pain in contrast to inflammatory pain in patients undergoing MTX treatment [5, 8, 12–14]. This in turn is immobilizing the patients to a severe extend [5]. Here, a specific radiological pattern has been identified, that relates to the lower extremity stress fractures associated with MTX treatment. More precisely, it has been characterized as stress fractures that take on a band- or meander-shaped appearance along the growth plate, referred to as epimetaphyseal osteolysis or band-like sclerosis [5].

As a treatment regime, first of all, stopping MTX treatment is the main approach presented in literature [12], yet the use of a bone-specific drugs has also been reported in some cases [5, 12, 13, 15] and may be of substantial importance since even after MTX discontinuation, the fracture healing may be delayed and new fractures may occur [12]. While different agents such as anabolic (teriparatide) and antiresorptive (bisphosphonate or denosumab) approaches, have been mentioned [4, 5, 16], today, a systematic comparison of the treatment options in a larger cohort is still missing.

As already stated in the title of the paper by Ragab et al. in 1970 [1], MTX osteopathy is frequently associated or confused with osteoporosis both in science and in everyday clinical practice [1, 2, 17]. The reasons for this confusion could be the multiple inadequate fractures accompanied by pain, the age-range of affected individual or a possibly densitometrical associated osteoporosis itself. Therefore, it is of great importance to differentiate MTX osteopathy from classic osteoporosis, which is also associated with repeated fractures and mobility restrictions [3, 5, 12], and to examine the treatment options in detail. Beyond that, since most of the today’s knowledge comes from case reports and the largest cohort reported encloses 34 cases [5], these two diseases are in need to be analyzed in a large cohort comparatively, in which the characterization is standardized, not differing between cases.

With this study we aim to compare a large cohort of patients with MTX osteopathy to those with osteoporosis and fractures assessing the therapeutic success in patients with MTX osteopathy, considering the impact of different therapeutic agents.

We hypothesize that MTX osteopathy differs significantly from osteoporosis showing a distinct clinical phenotype. Furthermore, we hypothesize that the response to treatment of patients suffering from MTX osteopathy also differs between different osteological therapeutic agents.

## Materials and Methods

### Patients

**Fig. 1:**
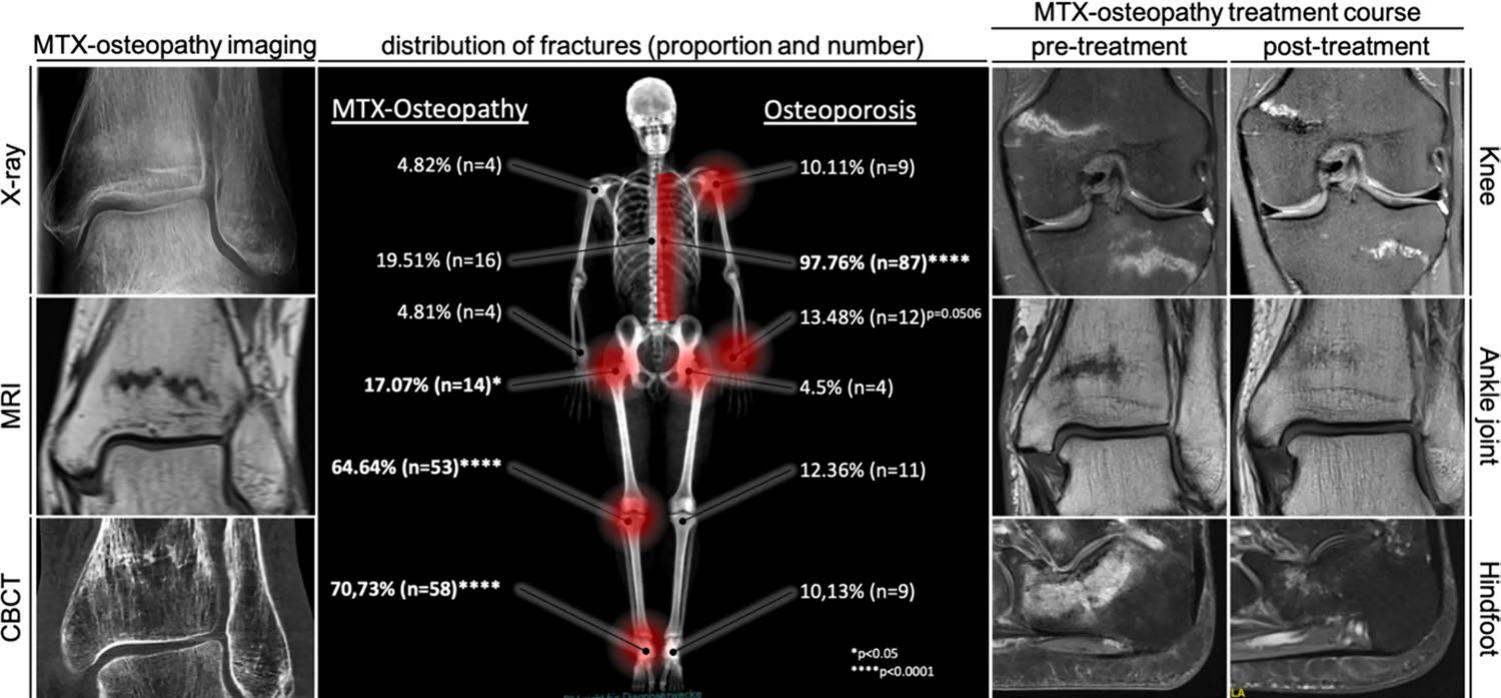
Comparative fracture occurrence for both groups: MTX osteopathy can be identified using different imaging modalities such as X-ray, magnetic resonance imaging (MRI) and computed tomography (CBCT) with a band- or meander-shaped appearance along the growth plate by means of densification or signal. Regarding skeletal distribution, MTX osteopathy patients do mainly exhibit fractures of the lower extremity with a significantly higher frequency of the proximal femur (p < 0.05), the distal femur and proximal knee (p < 0.0001) as well as the distal tibia, talus and calcaneus (p < 0.0001), indicated in red shading at the left side. Osteoporosis, in contrast, is mainly characterized by vertebral fractures (p < 0.0001) and fractures of the distal radius extremity (p < 0.0506). A bone specific therapy can significantly reduce the symptoms and ensure consolidation of the fracture with accompanying morphological improvement

Patients were evaluated as part of their clinical consultation between Jan. 2018 and Dec. 2022 at our outpatient clinic. Patients were screened according to their diagnosis and patients with MTX osteopathy diagnosed by an experienced physician were selected for the MTX group. Criteria for diagnoses were the typical fracture pattern (described above and in Fig. 1) and an active MTX treatment at first presentation. Special attention was given to the intake of MTX as well as the fracture pattern in the form of a meandering stress fracture; the location of the fracture within the skeletal system was explicitly not decisive. Patients were followed up for DXA for at least one year. Patients diagnosed with osteoporosis and at least one atraumatic/ osteoporotic fracture were selected for the osteoporosis group (OPO).

### Clinical Characteristics

The fracture status was assessed by means of a clinical interview and imaging that was requested or brought along. Vertebral fractures were assessed using vertebral fracture assessment (VFA) if no recent spine radiograph or MRI were available in accordance to Genant et al. [18] For this purpose, the patient was positioned laterally on the DXA device (Lunar iDXA, GE Healthcare, Madison, WI, USA) according to the manufacturer's instructions. Patients were positioned on their left side. In case of inability to lie on the left side, patients were positioned on the right side for VFA. Pain was assessed using a visual analogue scale (VAS). Restricted mobility was assessed in the MTX group using a numerical scale. The mobility restriction scale contained five levels: 0 = unrestricted mobility, 1 = restricted mobility, abnormal gait pattern without using walking aids, 2 = forearm crutches, 3 = need for a walker, 4 = wheelchair requirement. The mobility restriction scale was assessed both at the start of therapy and in the follow-up of the treatment course. Treatment involving an anabolic drug was grouped as anabolic treatment including teriparatide, DATA-scheme (denosumab in combination with teriparatide) and romosozumab, antiresorptive treatment was either bisphosphonate or denosumab. Basic (basic) treatment involves the supplementation of vitamin D and ensuring a daily calcium supply of at least 1000mg.

### Biochemical Assessment

Within clinical routine, analysis of the calcium homeostasis and bone turnover was carried out at our local laboratory (Institute of Clinical Chemistry and Laboratory Medicine, University Medical Center Hamburg-Eppendorf, Germany). Serum levels of calcium, phosphate, vitamin D (25-OH-D3), parathyroid hormone (PTH), and ALP (alkaline phosphatase) as well as urine calcium and creatinine were measured using the Atellica-Solution by Siemens. Bone formation markers such as BALP (bone-specific alkaline phosphatase) and osteocalcin were measured using the Liaison XL by Diasorin, while procollagen type 1 N-terminal peptide (PINP) and the bone resorption marker C-terminal telopeptide of type I collagen (ß-CTX) were measured using the Cobas E411 by Roche Diagnostics. Urine levels of deoxypyridinoline/creatinine (DPD) were measured using the Immulite-XP by Siemens.

### DXA Areal Bone Mineral Density (aBMD)

Areal bone mineral density (aBMD) was measured during routine clinical assessment according to the ISCD [19]. T-scores were calculated by the manufacturer’s software. The DXA measurement was carried out on a Lunar iDXA (GE Healthcare, Madison, WI, USA). Scans were performed at the lumbar spine (L1–4) and both proximal femora (femoral neck and total hip). The mean T-score of the spine (lumbar vertebral body L_1_–L_4_) and the lowest T-score of the total femur or femoral neck were selected. ´

### High-Resolution Peripheral Quantitative Computed Tomography (HR-pQCT) Measurements

High resolution peripheral quantitative computed tomography (HR-pQCT) was carried out on first-generation HR-pQCT equipment at 82 µm isometric voxel size (XtremeCT I, Scanco Medical AG, Brüttisellen, Switzerland) or second-generation HR-pQCT equipment at 60.7 µm isometric voxel size (XtremeCT II, Scanco Medical AG, Brüttisellen, Switzerland. Scanning was performed according to establish guidelines [20] by trained technicians at the nondominant radius and the contralateral tibia.

Evaluation of the scanned data sets were done by manufacturers evaluation program and scripts according to the manufacturer’s guidelines. All results were match first generation HR-pQCT scales by recalculation second generation results according to Manske et al. [21]. Prior to evaluation, data sets were check for motion artifact according to the manufacturers motion grading and scans with motion artifact above three were excluded from evaluation [20, 22]. Following structural parameters were considered for this study: trabecular bone volume to tissue volume ratio (BV/TV), trabecular thickness (Tb.Th) and trabecular number (Tb.N) as well as cortical thickness (Ct.Th).

### Muscular Performance and Balance Assessment

Measurements were conducted when the patient's clinical condition permitted. If immediate measurement was not feasible, assessments were carried out as soon as the patient's clinical status allowed. Follow-up measurements were conducted as part of the routine clinical examination for all consenting patients. In addition to specific parameters, an evaluation was made to determine the number of patients who, due to the effects of the therapy, became eligible for an assessment of muscle performance and balance, having been unable to participate in such examinations previously.

Muscle performance was measured by grip strength and chair raising test (CRT). The maximum grip strength was assessed using a hand-held dynamometer (Leonardo Mechanograph® GF, Novotec Medical, Pforzheim, Germany) when patients calmly seated with their arms resting on their thighs. Per arm, three measurements were taken (left/right), and the highest value of those was used for further evaluation. CRT was performed on a Leonardo Mechanograph® (Leonardo Mechanograph® GRFP STD, Novotec Medical, Pforzheim, Germany). The patients were instructed to sit on a bench and perform five cycles of standing up and sitting down as rapidly as they could. The force plate was used to record both the maximum force and the time taken per repetition.

Romberg posturography was utilized to evaluate balance, with the assistance of the Leonardo Mechanograph® GRFP for quantification. Patients were positioned on the force measurement platform, adopting a stance with feet together and arms extended at shoulder height. They were instructed to maintain a steady stance for ten seconds with their eyes open, assessing balance under visual control. Subsequently, the test was repeated with eyes closed to assess balance without visual input. The ground reaction force platform recorded the movement of the center of pressure over ten seconds for both conditions (eyes open and eyes closed). The corresponding path length (mm) was calculated following the methodology outlined by Simon et al. [23].

### Statistical Evaluation

The groups were first checked for normality, and depending on the question, parametric tests were used for comparisons when each group was normally distributed, and non-parametric tests were used for data that were not normally distributed. For follow-up data of the same patient, the paired t-test or Wilcoxon test for non-normally distributed data was used for a comparison of two time points. For simple two group comparison, students t-test or Mann–Whitney-U test for non-normally distributed data facilitated. For more than two groups, ANOVA with post-hoc testing using Šídák's multiple comparisons test was used for normally distributed data, and the Kruskal–Wallis test with Dunn’s multiple comparison test was used for data that are not normally distributed. Contingency testing for frequency analysis was carried out using Chi-squared testing. A p-value ≤ 0.05 was considered statistically significant.

Patients were stratified based on changes in the Visual Analog Scale (VAS) and mobility scale during therapy (ΔVAS > 3 for large improvements or ΔVAS ≤ 3 for small improvements; Δmobility > 1 for substantial mobility gains or Δmobility ≤ 1 for minimal gains). We then analyzed these groups to determine if significant improvements were associated with a specific treatment regimen. Comparisons between the large and small gain groups, across the three therapy types (anabolic, antiresorptive, and basic), were conducted using the Chi-square test. To investigate if the initial presentation status influences mobility gain or pain reduction, patients were stratified as previously described, followed by comparisons between mobility and pain values.

## Results

### Patient Characteristics

In this study, 83 patients with MTX osteopathy and 89 patients with osteoporosis have been included. No significant differences were detected regarding the patients age and height of the two cohorts (Table 1). Body weight and corresponding body mass index were significantly higher in the MTX group compared to the OPO group (both p < 0.005). The sex ratio was comparable in both groups. Mean dose of MTX was 17.33mg/week ± 4.67mg/week, mean treatment duration with MTX prior to first presentation was 127month ± 93.7month, none of the OPO groups received MTX. 47 MTX patients received corticosteroids with a mean dose of 6.16mg/d ± 4.96mg/d while only two OPO patients received corticosteroids (one 3mg/d and one 5mg/d).

**Table 1:** Demographic, densitometric and laboratory assessment of MTX and OPO group at baseline: Values out of the normal range and significant p-values are printed in bold

			MTX		OPO		
			Mean	± SD	Mean	± SD	Control vs. MTX
n			83		89		
Parameter	Unit						p-value
Demographics							
Age	Years		68.95	8.79	69.85	8.26	0.49
Height	m		1.65	0.07	1.64	0.08	0.22
Body weight	kg		69.14	14.14	62.95	11.55	**< 0.005**
BMI	kg/m2		25.22	4.46	23.42	3.48	**< 0.005**
Sex	f:m		75:8		80:9		0.92
Disease frequency							
dOPO	%		45.8		100		
RA	%		70.9		1.1		
PA	%		14.0		0.0		
SLE	%		3.5		1.1		
ORD	%		11.6		2.3		
Densitometry and fracture							
VF	%		19.5		97.8		**< 0.0001**
T-score spine	SD		− 1.96	1.45	− 3.08	1.38	**< 0.0001**
T-score hip	SD		− 2.45	1.01	− 2.72	0.96	**0.04**
Laboratory assessment		Range					
Calcium	mmol/l	2.08–2.65	2.35	0.13	2.38	0.13	0.3
Phosphate	mmol/l	0.78–1.65	0.98	0.18	1.03	0.17	0.06
AP	U/l	46–116	92.15	29.11	90.77	26.58	0.94
BGLAP	µg/l	12-52.1	18.81	9.02	21.39	9.53	**0.05**
Vit. D3	µg/l	> 30µg/l	39.93	13.45	35.53	11.89	**0.04**
bAP	µg/l	5.5–22.9	18.37	8.57	14.73	6.1	**0.02**
PINP	µg/l	32.0–123.0	63.4	41.89	84.85	54.32	0.29
β-CTX	µg/l	0.238–1.019	0.26	0.18	0.42	0.29	0.09
PTH	µg/l	18.4–80.1	62.06	30.14	59.79	25.51	0.8
DPD	nmol/mmol	< 7	**7.95**	2.67	**8.66**	3.23	0.37

*BMI *body mass index, *f* female, *m* male, *dOPO* densitometric osteoporosis (according to WHO, T-score ≤ -2.5), *RA* Rheumatoid arthritis, *PA* psoriatic arthritis, *SLE* systemic lupus erythematosus, *ORD* other rheumatoid disease, *VF* vertebral fracutres, *SD* standard deviation, *AP* alkaline phosphatase, *bALP* bone-specific alkaline phosphatase, *BGLAP* osteocalcin, *Vit. D*_*3*_ 25-hydroxyvitamin D_3_, *PTH* parathyroid hormone, *DPD* deoxypyridinoline, *β-CTX* β-crosslabs, *PINP* procollagen type 1 N-terminal propeptide, *SD* standard deviation

Underlying disease frequencies are presented in Table 1. Other rheumatoid diseases (ORD) include polymyalgia rheumatica, adult onset Still’s disease, mixed connective tissue disease, undifferentiated HLA B27 negative spondylarthritis, Behcet’s disease, Felty syndrome, sarcoidosis or posterior uveitis. In contrast, in the OPO group 95.51% had no rheumatoid disease (Table 1).

### Distribution of Fractures in OPO vs. MTX

Comparing the frequency of all fractures in both groups, a significantly higher frequency was detected for the lower leg regions in case of MTX osteopathy. No significant differences were detected between both groups for the upper extremity but a borderline significance (p = 0.0506) indicated a tendency towards higher frequencies of distal radius fractures in the OPO group. A significantly larger portions (p < 0.0001) of patients did exhibit vertebral fractures in the OPO group (97.76%) compared to the MTX group (19.51%). Taken together, the fracture pattern of MTX-patients differs significantly from typical osteoporotic fractures as depicted in Fig. 1. In total 149 fractures were seen in the MTX group and 132 in the OPO group.

### Pre-treatment Presentation OPO vs. MTX

Laboratory assessment (Table 1) of the two groups prior to treatment initiation did not show significant differences for the following parameters: calcium, phosphate, AP, PINP, β-CTX, PTH and DPD/creatinine. Significantly higher serum levels were measured for vitamin D in the MTX group compared to OPO (39.93µg/l ± 13.45µg/l vs. 35.53µg/l ± 11.89µg/l, p < 0.05) and BGLAP was lower in the MTX group compared to OPO (p ≤ 0.05) while bAP was higher in the MTX-group (p < 0.05). Stratifying the MTX group for glucocorticoid (GC) treatment or not did not indicate any significant differences between the groups in bone laboratory parameters. 48.08% of the MTX group had an osteoporosis by densitometric definition (T-score ≤ -2.5). Stratification for osteoporosis in the MTX-group revealed significant differences only in the DPD measurements (MTX_OPO_: 7.18 nmol/mmol ± 2.41nmol/mmol, MTX_no OPO_: 8.61 nmol/mmol ± 2.75 nmol/mmol, p < 0.037). Age did also not differ significantly between the subgroups (MTX_OPO_ and MTX_no OPO_).

**Fig. 2:**
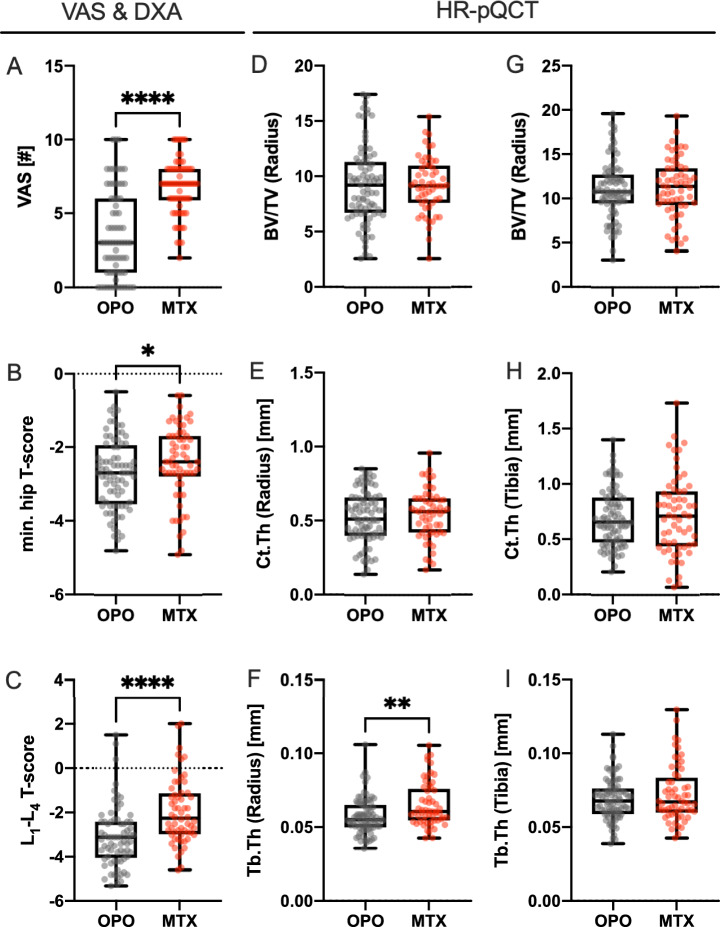
Pre-treatment pain levels and bone measures: A significantly (p < 0.0001) higher pain level was detected in the MTX group (**A**). Minimal T-score of spine and hip (**B**) and spinal mean T-score (**C**) were significantly higher in the MTX group, yet generally reduced compared to a healthy reference group. *min. hip T-score* minimal T-score at hip, *L*_*1*_*-L*_*4*_* T-score* mean T-score at the spine, *BV/TV* bone volume to tissue volume, *Ct.Th* cortical thickness, *Tb.Th* trabecular thickness, *p < 0.05, **p < 0.01, ****p < 0.0001

Comparing the pain levels (Fig. 2a) of the two groups prior to treatment start exhibits higher mean pain levels in case of MTX osteopathy compared to OPO (MTX: 6.75 ± 1.86, OPO: 3.62 ± 2.95, p < 0.0001). No significant differences were observed for pain levels stratified by GC treatment in the MTX group. Mean minimal T-score selected from femoral neck and total femur was significantly lower in the OPO-group compared to MTX (Fig. 2b; MTX: − 2.38 ± 1.10, OPO: − 2.72 ± 0.96, p = 0.024) with the analogue pattern in the mean spinal T-score (Fig. 2c; MTX: − 1.96 ± 1.45, OPO: − 3.08 ± 1.38, p < 0.0001) and no differences stratifying for GC treatment in the MTX group. When stratifying mobility in the MTX group for GC treatment, patients receiving GC had significantly pronounced mobility restrictions than others (with GC: 2.38 ± 1.23, without GC: 1.73 ± 0.98, p = 0.039). For HR-pQCT parameters (Fig. 2d–i), no significant differences were detected between both groups except a significantly lower radial Tb.Th in case of OPO compared to MTX (MTX: 0.066mm ± 0.015mm, OPO: 0.0577mm ± 0.012mm, p = 0.0013).

### Treatment Effects OPO vs. MTX

Among OPO patients, 76.4% received antiresorptive drugs, 21.35% were treated with anabolic drugs, and 2.24% used only vitamin D and calcium due to refusal of bone-active drugs. In contrast, for MTX osteopathy patients, 30.12% were treated solely with antiresorptive drugs, 45.78% with anabolic drugs, 14.46% used only vitamin D and calcium, and 9.64% refused all treatments. Additionally, 83.13% of MTX osteopathy patients discontinued MTX after diagnosis, while 16.87% continued its use. 24 of the MTX patients received bisphosphonates before their first visit to our outpatient clinic, with no other bone-specific treatments administered.

**Fig. 3:**
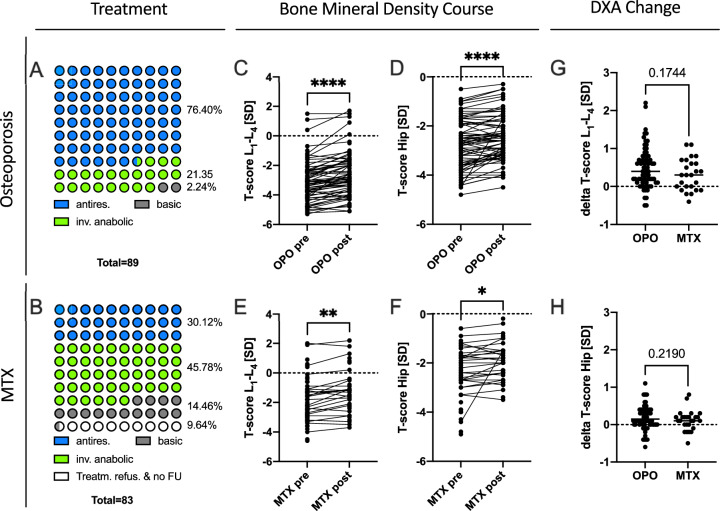
Treatment regime and DXA follow-up: Distribution of different treatment regimes for the OPO (**A**) and MTX group (**B**) Treatment regimens of OPO patients. **A** consisted predominantly of antiresorptive agents while in the MTX-group. **B** the majority received anabolic medications. **A** significant increase in both, spinal and hip T-scores was observed in the OPO group (**C**–**D**) as well as in the MTX group **E, F**). No differences were found in the change of the T-score increase for the spine (**G**) or hip (**H**) between the two pooled groups, yet at a differing mean therapy regime. *p < 0.05, **p < 0.01, ****p < 0.0001

Mean follow-up time for DXA measurements did not differ significantly between OPO and MTX group (MTX: 12.66 month ± 1.80 month, OPO: 12.03 month ± 4.56 month, p = 0.82). Mean bone mineral density of the OPO group increased significantly in the spine (Fig. 3c, p < 0.0001) and in the hip (Fig. 3d, p < 0.0001) in the course of therapy. Correspondingly, a significant aBMD increase in the MTX group was measured comparing pre-treatment DXA results and follow-up measurements for spine (Fig. 3e) and hip (Fig. 3f).

Comparing OPO and MTX patients, there were no significant differences in T-score gains for spine and hip (Fig. 3G, H). Stratifying for densitometric osteoporosis (according to WHO, T-score ≤ -2.5) or not in the MTX group revealed a mean T-score of − 3.30 ± 0.67 in the MTX_OPO_ and a mean of − 1.63 ± 0.51 in the MTX_no OPO_ group (p < 0.0001). Comparing the change in laboratory results pre and post treatment, no significant differences were found in the subgroups.

### Treatment Effects on Clinical Parameters in MTX Osteopathy

**Fig. 4:**
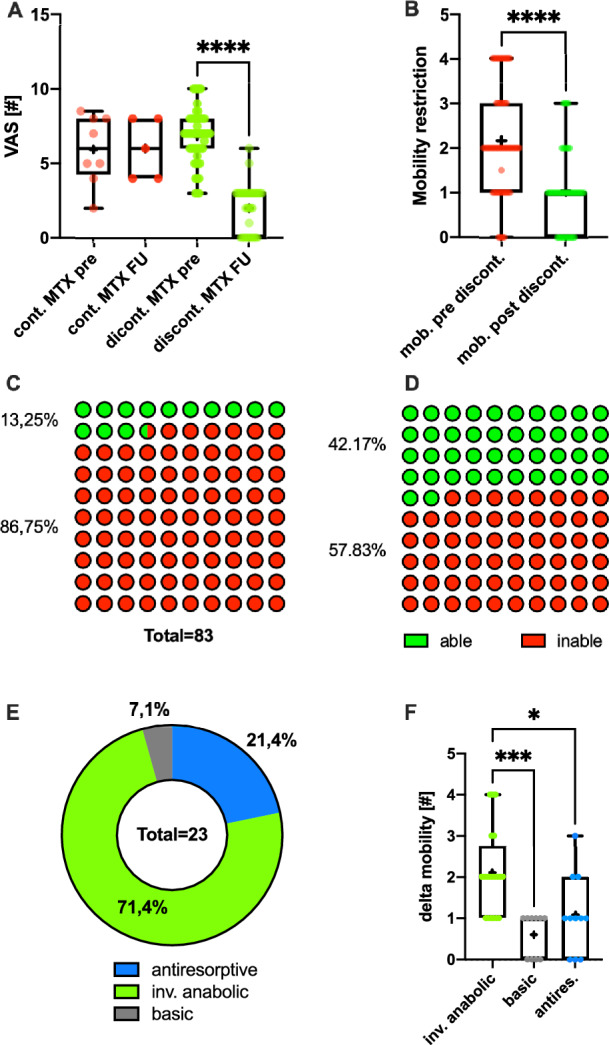
Treatment effects on clinical outcomes in MTX osteopathy patients: Patients not discontinuing MTX therapy did not exhibit lower pain levels in the course of therapy while patients discontinuing MTX profited from MTX discontinuation (**A**). Furthermore, mobility restriction was decreased when treating patients who discontinued MTX with bone active drugs (**B**). Out of all patients at the first presentation, only a small portion was able to perform muscular performance testing and Romberg testing (**C**), after treatment significantly more (p = 0.0001) patients were able to perform the testing in the follow-up (**D**). Out of the patient that gained ability to do testing, significantly more patients received anabolic treatment compared to the full therapy cohort (**E**). When comparing mobility gain, patients with anabolic treatment gained significantly more mobility than others (**F**). *VAS* visual analogue scale, *cont. pre* continued MTX treatment, measurement at first presentation, *cont. post* continued MTX treatment, follow-up measurement, *discont. pre* stopped MTX treatment, measurement at first presentation, *discont. post *stopped MTX treatment, follow up measurement, *inv. anabolic* treatment involves the use of an anabolic drug; *p < 0.05, **p < 0.01, ***p < 0.0005, ****p < 0.0001

Treatment effects on pain level and mobility were re-quantified in sub-groups of patients suffering from MTX-osteopathy. No difference in pain levels was observed in the follow-up in patients continuing MTX (VAS first presentation: 5.94 ± 2.31 vs. VAS follow up: 6.00 ± 2.00; p > 0.05; Fig. 4A). When comparing the mean VAS level pre therapy and in follow up in patients that discontinued MTX, a significant drop in pain levels was observed (pre: 6.82 ± 1.77 vs. follow up: 2.00 ± 1.55, p < 0.0001; Fig. 4A). No differences were found in VAS change when stratifying MTX patients by GC treatment. Mobility restriction quantification at first presentation (2.16 ± 1.19) and follow up (1.04 ± 0.87) did indicate significantly (p < 0.0001) reduced values at initial presentation corresponding to significantly improved mobility for treated patients who discontinued MTX (Fig. 4B). No significant differences were found for patients change in mobility stratifying for GC treatment in the MTX group.

Out of 83 patients diagnosed with MTX-osteopathy, only 13.25% had been able to perform a muscle performance (CRT and GF) and Romberg posturography at initial presentation (Fig. 4C). In the follow up (Fig. 4D), significantly more patients (42.17%) were able to perform the testing (p < 0.0001). Considering the treatment distribution of all treated patients, 16% received vitamin D and calcium only, 33% received antiresorptive medication and 51% received an anabolic drug.

Among patients who could perform muscular and Romberg tests during therapy but not at the start, 7.1% received vitamin D and calcium, 21.4% were on antiresorptive treatment, and 71.4% received anabolic drugs (Fig. 4E). Chi-square testing showed a significant (p = 0.009) higher success rate in performing these tests in the course of therapy for those on anabolic therapy. Additionally, patients on anabolic therapy had significantly greater mobility gains compared to those on antiresorptive therapy (p < 0.0005) or vitamin D and calcium only (p < 0.05) (Fig. 4F). No significant differences were found in treatment time until meaningful clinical improvement among the groups.

### Choice of Treatment in MTX

When comparing therapies based on pain improvement (ΔVAS > 3 for large or ΔVAS ≤ 3 for small), no significant difference was found (p = 0.52). However, there was a significant difference in mobility gains (p = 0.02). Specifically, a higher proportion of patients who experienced substantial mobility improvement (Δmobility > 1) was treated with anabolic therapy, whereas those with minimal mobility gain (Δmobility ≤ 1) were less likely to receive anabolic treatment.

**Fig. 5:**
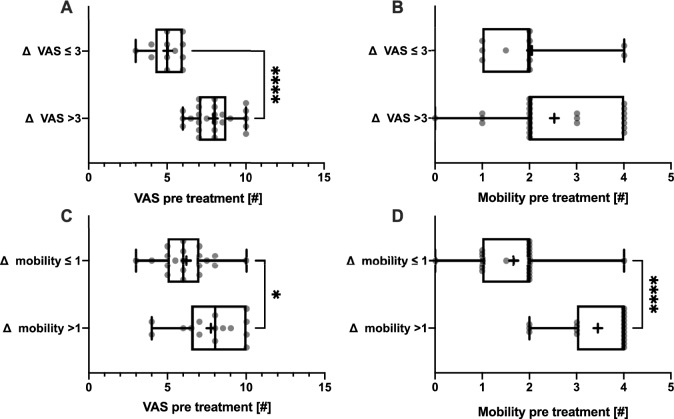
Treatment success dependent on first presentation: Significantly lower baseline pain (**A**) values were measured for patients with VAS improvement of 3 or less. No difference was seen in mobility restriction (**B**). Significantly lower baseline pain (**C**) and mobility restriction (**D**) values were measured for patients with mobility improvement of 1 or less. *VAS* Visual Analog Scale for pain; *p < 0.05, ****p < 0.0001

Patients with a more pronounced decrease in pain had significantly higher baseline VAS scores (7.92 ± 1.32; p < 0.0001) compared to those with minimal improvement (ΔVAS ≤ 3; Fig. 5A). This difference remained significant after adjusting for baseline VAS (ΔVAS > 3: 0.79 ± 0.19, ΔVAS ≤ 3: 0.39 ± 0.15; p < 0.0001). Therefore, patients with higher pain levels at the beginning of the therapy have greater improvements even after normalization to their initial VAS score. No significant difference in mobility restriction was observed based on pain improvement (Fig. 5B).

Patients who showed greater improvements in mobility (Δ mobility) initially had significantly higher pain scores (p < 0.05) compared to those with minimal mobility gains (Fig. 5C). Similarly, individuals with more pronounced mobility improvements had higher baseline mobility restrictions (Fig. 5D). The difference in baseline mobility remained significant after normalization to the baseline mobility value (Δmobility > 1: 0.76 ± 0.21, Δmobility ≤ 1: 0.43 ± 0.32; p < 0.0005).

## Discussion

In this study, we examined 83 patients diagnosed with MTX osteopathy, compared them to age-matched osteoporosis patients with atraumatic vertebral fractures, and tracked their progress over time under specific therapy. MTX osteopathy patients exhibit significantly more stress fractures in the lower extremities, particularly near the joints, aligning with previously described pattern [3, 5]. Notably, irrespective of the chosen osteological drug, pain relief is observed in MTX osteopathy patients only with the discontinuation of MTX therapy, underlining the pivotal role of this therapeutic measure. Additionally, patients with early remobilization did more frequently receive osteoanabolic therapy.

MTX patients, which were mainly female, are characterized by a higher body weight and higher BMI in comparison to OPO patients, in line with literature [24], which may be a sign of their underlying rheumatological disease and the associated restriction of movement limiting their physical activity. No differences were seen between the two groups with respect to sex or age. In line, MTX patients with GC treatment had higher mobility restrictions than others. There is a significantly higher frequency of MTX osteopathy manifestations in the lower extremity showing a characteristic fracture appearance (Fig. 1) compared to an osteoporosis group with atraumatic vertebral fractures. This highlights the relevance of the fracture site and appearance for suspicion of MTX-osteopathy, matching the published literature [3]. Specifically, over 70% of MTX patients have stress fractures of the upper or lower ankle joint and almost 2/3 have stress fractures of the bones forming the knee. This accumulation clearly indicates the primary manifestation of MTX osteopathy in the weight bearing bones of the lower extremity. Notably, to our knowledge the presented size of our cohort from one clinical center does outperform not only several cohorts describing MTX osteopathy but also the size of assembled review cohorts[3] composed of several individual publications, increasing the evidence of the lower extremity to be the area of primary manifestation.

Blood sampling indicates a shift of bone metabolism with a decreased osteoblast action mirrored by lower BGLAP but a higher bAP level. This is of particular interest, since MTX patients did present with stress fractures, for which elevated levels of bone formation by means of alkaline phosphatase [25–27] and BGLAP [27, 28] in response to the fractures respectively fracture healing is known and expected. Of note, since GC treatment may introduce a low turnover, stratification for GC treatment in the MTX did not indicate any differences, suggesting the low turnover to be independent of GC and mainly caused by MTX.

Bone resorption was above reference yet did not differ from OPO patients and was only single-digit compared to usually double-digit values in fractures [27]. This aspect might point to a low turnover bone metabolism (with respect to an expected high turnover in case of a present stress fracture) and thereby inability to initiate adequate fracture healing in case of MTX treatment. This may be caused by MTX, known to decrease metabolic processes by its very own drug mechanism [29]. The sufficient vitamin D level in both groups may be explained by regular medical care and the significantly higher levels in the MTX group by a more frequent medical health care consultation for those with chronic illnesses.

Pain levels in MTX patients are significantly higher than those in OPO patients, mainly explained by the presence of non-silent fractures in the weight-bearing lower extremity in MTX osteopathy as opposed to the often-silent vertebral fractures in OPO. While bone mineral density (BMD) is negative in all MTX osteopathy patients, the T-scores in the OPO group are notably lower. This indicates that the primary factor for fracture occurrence in MTX might not be aBMD itself but rather metabolic factors as described above. Nevertheless, it's worth noting that many patients meet the WHO definition of osteoporosis [30], where osteoporosis is known to be the most frequent bone disease in rheumatoid patients [31]. Since 55% of our MTX patients do not exhibit osteoporosis, it is evident that osteoporosis is not the primary factor driving MTX osteopathy. In fact, given that all MTX patients have an osteoporosis-favoring rheumatoid disease, MTX patients without osteoporosis had relatively favorable mean T-scores (− 1.6) considering the cohort's age and underlying rheumatoid disease. However, osteoporosis might be a risk factor for developing MTX-osteopathy since it was present in 45% of the respective patients. HR-pQCT imaging did only exhibit a lower Tb. Th in the radius of OPO patients compared to MTX, thus no major differences in bone microstructure were detected, but site and thus loading specific changes may be relevant.

While the largest portion of OPO patients received an antiresorptive agent, most patients with MTX osteopathy were treated with an anabolic agent. This can be attributed to the particularly low bone turnover observed in the MTX group in response to the stress fractures. Furthermore, MTX osteopathy is linked to fracture healing issues, with teriparatide being the only treatment supported by data for such cases [32, 33]. Additionally, at the time of inclusion, national guidelines for osteoporosis treatment favored antiresorptive therapy as the first-line option for osteoporosis. Hence, T-scores did significantly increase in the course of therapy for both, MTX and OPO. Notably, there was no significant difference of bone mass gain between OPO and MTX even considering the MTX group to exhibit a larger portion of anabolic treatment. While anabolic treatment options are known to decrease fracture occurrence significantly more than antiresorptive agents [34], and increase BMD after antiresorptive treatment[35], our finding is of particular interest and potentially points to the low responsiveness of the MTX affected bone. On the other hand anabolic treatments such as teriparatide are known to affect the spine more compared to antiresorptive treatment with regard to BMD gain [36]. But this pattern was not observed in the current study comparing the treatment effects of the OPO and MTX group (Fig. 3). Furthermore, 45% of MTX patients have both MTX osteopathy and osteoporosis. This highlights the need to not only stop MTX treatment as the primary approach but also to initiate bone-specific therapy, preferably anabolic treatment, based on our data. This applies not only to those with densitometric osteoporosis (T-score ≤ − 2.5) but also to most of the patients with MTX-associated stress fractures. In addition, the large proportion of OPO patients may indicate that osteoporosis is a risk factor for the development of MTX osteopathy.

After comparing the general treatment response in both groups, we have specifically focused on the treatment effects on MTX patients. Pain reduction was only perceived in case of MTX discontinuation (Fig. 4a), as described by others [4, 16, 37], yet fracture healing may remain limited [12]. After treatment, patients mobility was significantly improved as well as a significantly higher portion of patients was able to perform muscular performance and balance assessment, indicative of treatment success beyond pain relief. In particular, patients exhibit significantly larger improvements in their mobility restriction when receiving a therapy involving an anabolic drug.

The therapy-specific difference was analyzed in more detail accordingly. There were no frequency deviations between patients with large and small improvements in pain depending on the therapeutic agent. This means that the reduction in pain seems to be independent of the choice of the therapeutic agent but relies mainly on MTX discontinuation (Fig. 4a). However, when comparing the frequency of therapy choice of patients with large improvements in mobility to small improvements, significant frequency deviations were seen involving an anabolic drug exhibiting better improvements.

From our results, it can be conducted that patient’s mobility restriction levels upon first clinical presentation do not determine the improvement in pain (Fig. 5b) yet pain levels do. On the other hand, pain and mobility restriction levels do determine the patients pain relieve (Fig. 5c) and gain in mobility (Fig. 5d) even after adjustment for primary mobility levels. Stratification for GC treatment did not indicate differences in mobility gain by treatment between patients with or without GC treatment, even given the higher restrictions in the GC subgroup at start of the therapy.

This study has limitations. Due to its retrospective design, not all data points were available for follow up measurements, yet this is the largest cohort from one specific outpatient clinic thereby limiting variations in alertness of the physicians treating the patients as well as examinations chosen and variations in such and thereby guarantee a high level of reliability compared to datasets combined of case reports. Furthermore, we are not able to directly link MTX and the clinical pattern, yet today this specific pattern is exclusively observed in patients treated with MTX and is supported by experimental evidence [38–40]. Thirdly, since rheumatoid diseases are challenging with respect to treatment and require tailored therapy regimes, interactions may be present, yet reporting the by far largest cohort of MTX osteopathy, such interactions may be less prominent due to the number of patients. Fourth, subgroups of treatment are small, compared to the full cohort, limiting the statistical accuracy due to interacting parameters. To fully address the above-mentioned points, longitudinal studies are needed. Furthermore, reported pain levels may be influenced by timepoint of measurement, fracture location (OPO primarily in the spine, MTX primarily at the lower extremity), the underlying rheumatoid disease and possibly taken analgesics. Patients were not randomized with respect to MTX discontinuation in this retrospective study. But it is questionable whether such a randomization would be reasonable given the already existing body of literature. Importantly, the diagnosis of MTX osteopathy cannot rely solely on the fracture site. Radiological confirmation is essential, with a typical MTX osteopathy diagnosis requiring the identification of a meander-shaped, band-like sclerosis region indicative of a stress fracture on X-ray, CT, or preferably MRI. Thus, such diagnostics should be considered in respective patients.

## Conclusion

Taken together, we present data of a single center study including 83 patients suffering from MTX osteopathy compared to osteoporosis patients. Main clinical sign is a symptomatic stress fracture at the lower extremity with a distinct appearance, mostly at the knee or ankle joint. MTX-patients exhibit a low turnover bone metabolism in the light of an acute stress fracture. They do exhibit reduced bone mineral density yet less pronounced than in case of OPO. Pain relieve is perceived after MTX discontinuation and bone specific treatment increases mobility of the patients most effectively by involving anabolic agents.

## Data Availability

The data underlying this article will be shared upon reasonable request in compliance with national data protection regulations.
